# Implementation and Applications of Artificial Intelligence in Nutrition: A Systematic Review of Use in Practice and Research

**DOI:** 10.3390/nu18091340

**Published:** 2026-04-24

**Authors:** Celia Fabiola Vásquez-García, María Elizabeth Tejero, Marlen Naranjo-Martínez, Alexa Zagorin-Djaddah

**Affiliations:** 1 Tecnologico de Monterrey, Campus Santa Fe, Ciudad de Mexico 01389, Mexico; fabiola.vasquez@tec.mx(C.F.V.-G.); a01783992@tec.mx (A.Z.-D.); 2 Laboratorio de Nutrigenética y Nutrigenómica, Instituto Nacional de Medicina Genómica (INMEGEN), Ciudad de Mexico 14610, Mexico; etejero@inmegen.gob.mx

**Keywords:** artificial intelligence, clinical nutrition, public health, precision nutrition, nutrition interventions

## Abstract

**Background:** Artificial intelligence (AI) is increasingly incorporated into nutrition research and practice; however, the extent of its clinical integration and impact on health outcomes remains unclear. This systematic review evaluated how AI-based systems have been implemented in human nutritional interventions and summarized reported outcomes. **Methods:** PubMed, Scopus, Google Scholar, SpringerLink, JMIR, and MDPI were searched from January 2020 to March 2025 (search completed in March 2025). Randomized controlled trials and prospective or retrospective cohort studies published in English or Spanish were included if they evaluated AI-driven nutritional interventions in human populations and reported health-related outcomes. Risk of bias was assessed using RoB 2 and ROBINS-I. A qualitative synthesis was performed. **Results:** Sixteen studies involving 10,863 participants were included. Most were randomized controlled trials targeting metabolic disorders, particularly type 2 diabetes and obesity. Eleven studies evaluated metabolic outcomes, including HbA1c, body weight, fat mass, lipid levels, and insulin resistance indices. Six studies assessed gastrointestinal symptom severity scores, and two examined quality-of-life or patient-reported outcomes. Several trials reported short-term improvements favoring AI-supported interventions in glycemic control, weight reduction, and symptom severity. However, effects were heterogeneous and often observed within multimodal programs, limiting attribution of outcomes solely to the AI component. **Conclusions:** AI integration in nutrition remains in an early phase of clinical implementation. Although preliminary findings suggest potential benefits, interpretation should be cautious given methodological heterogeneity and moderate-to-high risk of bias across studies. Larger, rigorously designed investigations are required to determine sustained clinical effectiveness.

## 1. Introduction

Nutritional science holds a central place among scientific disciplines due to its direct impact on human development [1]. It encompasses the processes through which nutrients are extracted from food and utilized for the optimal functioning of cells, tissues, organs, and bodily systems [2]. Adequate nutritional intake is essential for growth, disease prevention, and the maintenance of quality of life, as most health conditions have an intrinsic relationship with diet [3]. Conversely, malnutrition, whether due to deficit or excessive intake—breaks this balance and results in an inefficient utilization of nutrients. [4,5] This discipline has undergone a significant transformation, driven by advances in scientific research and technological support. Currently, Artificial Intelligence (AI) has expanded into various fields of health sciences, including dietetics. However, the term “AI” is frequently used in an imprecise manner, often encompassing a broad range of digital health technologies that do not necessarily exhibit intelligent behavior. In this review, AI systems are considered models and a class of computational systems capable of learning from data, adapting their internal models, and generating outputs or decisions that are not fully pre-specified by human-defined rules. This definition aligns with widely accepted conceptualizations of AI applications as systems that emulate aspects of human cognition—such as pattern recognition, prediction, and decision-making—through data-driven learning processes rather than static logic [6,7].

This distinction is particularly relevant in the field of nutrition, where many digital tools—such as food logging applications, rule-based dietary calculators, or static decision trees—are often labeled as “AI” despite operating through predefined algorithms and expert-coded thresholds. While these digital or rule-based systems play an important role in nutrition practice, they lack the core characteristics of AI, namely the ability to iteratively learn from large and heterogeneous datasets, update predictions over time, and improve performance without explicit reprogramming.

In contrast, AI-based nutrition systems typically rely on machine learning or deep learning approaches that integrate multiple data streams—such as dietary intake, anthropometrics, biomarkers, lifestyle behaviors, or microbiome profiles—to generate personalized recommendations, forecast outcomes, or support clinical decision-making. These systems are designed to evolve as new data becomes available, allowing for dynamic adaptation to individual responses and contextual changes. Establishing this conceptual boundary is essential to accurately assess the current state of AI implementation in nutrition and to avoid conflating emerging intelligent systems with conventional digital health tools.

One of the main benefits of this technology is its ability to analyze the considerable amounts of data generated in the nutrition field [8]. Through the examination of large databases, it is possible to identify and forecast health concerns, an ability that becomes especially valuable considering the increasing prevalence of nutrition-related diseases and the burden caused by poor eating habits, malnutrition and obesity [9]. Furthermore, AI can allow healthcare providers to implement a more personalized approach to nutrition that considers factors like a person’s genetics, metabolism, microbiome, and lifestyle [10]. AI is also improving the understanding of the relationship between food and health. Additionally, users can now have support via apps that offer continuous feedback, monitor dietary intake, or motivate them to make healthier choices [11]. Thus, AI may be used for analysis of complex data for research purposes. In addition, it may support diagnosing, monitoring, or treatment systems used by healthcare professionals or patients.

Considering that the regulatory framework is still under development and that multiple ethical considerations remain regarding the implementation of these tools, most AI applications in nutrition are still in the developmental stage [12,13,14]. This evolving and often fragmented landscape has contributed to a limited understanding of which tools have been implemented in clinical practice, leading to an imprecise view of their real-world applications. Hence, the present review aims to address this knowledge gap, as a clear understanding of existing implementations is essential for accurately assessing how AI is currently being used to enhance patient care in nutrition. Such clarity will also help identify priority areas for the development of new tools and guide regulators in determining which aspects should be addressed first in future legislation to ensure safe, effective, and ethical integration of AI into nutritional practice. As the development and deployment of AI-based tools continue to evolve at a rapid pace, gaining a clear understanding of both the current landscape and future directions is essential for researchers working in this field.

The rapid expansion of technology-enabled nutrition tools has led to an increasingly broad use of the term “artificial intelligence” to describe heterogeneous computational systems. In the health sciences literature, AI may refer to adaptive data-driven models such as machine learning or deep learning, but it is also frequently used to denote rule-based expert systems or automated digital platforms [15,16,17]. This conceptual ambiguity complicates the evaluation of the true level of algorithmic sophistication and clinical integration of AI-based interventions in nutrition. Clarifying these distinctions is therefore essential to accurately assess the current maturity and contribution of artificial intelligence in human nutritional care.

This distinction was not intended to retrospectively exclude studies, but to enable structured analytical interpretation of the spectrum of technologies labeled as AI in clinical nutrition research.

Recent reviews have provided valuable and comprehensive overviews of artificial intelligence (AI) applications in nutrition and health, outlining the technological landscape and highlighting emerging opportunities in the field [18,19,20]. The present systematic review seeks to further advance the field by focusing specifically on AI-based interventions that have been implemented and evaluated in human populations.

Rather than revisiting the broad spectrum of potential applications, this review aims to examine the degree of real-world and clinical integration of AI systems in nutrition, with particular attention to the transparency of algorithmic reporting, the level of technical detail provided, and the practical context in which these systems are deployed. By exploring the nuances between different forms of AI, ranging from adaptive learning systems to rule-based digital tools, this work contributes to a clearer understanding of the current maturity of AI in nutrition and its evolving role in patient-centered nutritional care.

## 2. Materials and Methods

This systematic review was designed and reported in accordance with the PRISMA 2020 statement and checklist [21].

### 2.1. Research Question and Objectives

This review aimed to systematically identify and characterize nutritional interventions incorporating artificial intelligence components, and to examine the nature and reporting of associated health outcomes.

The guiding research question was:

To what extent have artificial intelligence tools been integrated into nutritional interventions in human populations, and what health-related outcomes have been reported?

### 2.2. Search Strategy and Information Sources

The identification of relevant literature on artificial intelligence in nutrition was conducted using multiple complementary sources grouped according to their functional role.

Structured Boolean search strategies were applied in the electronic bibliographic databases of PubMed and Scopus. The complete database-specific Boolean strings and the number of records retrieved per query are presented in (Supplementary Table S1).

Additional keyword-based searches were performed in the academic search engine of Google Scholar, as detailed in (Supplementary Table S2). Targeted searches were also conducted within publisher platforms (SpringerLink, JMIR, and MDPI), with the corresponding queries and yields reported in (Supplementary Table S3).

Citation-network exploration was further undertaken using Connected Papers and Research Rabbit. In Connected Papers, selected review articles were introduced as seed papers to explore similarity-based citation networks. In the Research Rabbit, keyword-initiated queries were used to examine related citation clusters. These procedures and their respective yields are described in (Supplementary Table S4).

### 2.3. Search Execution and Record Retrieval Approach

Searches were conducted sequentially within each source, acknowledging differences in indexing structure and interface functionality across platforms. Records were reviewed according to platform-defined relevance ranking and downloaded when considered aligned with the thematic scope of artificial intelligence applications in nutrition.

Within each query–source pair, screening proceeded until subsequent results appeared progressively less relevant to the predefined focus of the review. Consequently, the number of records retrieved varied across platforms and search inputs. The exact yield for each query–source combination (n = x) is reported in (Supplementary Tables S1–S4).

To enhance methodological consistency, screening within each query followed platform-defined relevance ranking and continued until thematic saturation was observed, reducing the likelihood of arbitrary truncation.

All records identified through this process were consolidated prior to deduplication and subsequently assessed according to the predefined eligibility criteria.

### 2.4. Eligibility Criteria and Study Selection Process

The present study applied the PICOS (Population, Intervention, Comparison, Outcomes, and Study Design) framework to define eligibility criteria, following established evidence-based methodological guidance for systematic reviews [22]. The PICOS structure was used to ensure transparency, reproducibility, and conceptual alignment between the research question and study selection (Table 1).

The review focuses on original research (clinical trials and cohort studies) assessing AI-driven nutritional interventions in humans. Due to the rapid evolution of this technology, the search was limited to peer-reviewed articles in English or Spanish published from January 2020 to March 2025.

### 2.5. Study Selection Procedure

A total of 796 records were identified from databases and other sources. All records were imported into Mendeley for duplicate removal. After removing 122 duplicate records, 674 records remained for screening. Two reviewers independently screened the records according to the predefined eligibility criteria. A total of 286 records were excluded at this stage. Subsequently, 388 reports were sought for retrieval; eight reports were not retrieved. The remaining 380 reports were assessed for eligibility according to the PICOS framework and operational definition of artificial intelligence.

**Figure 1:**
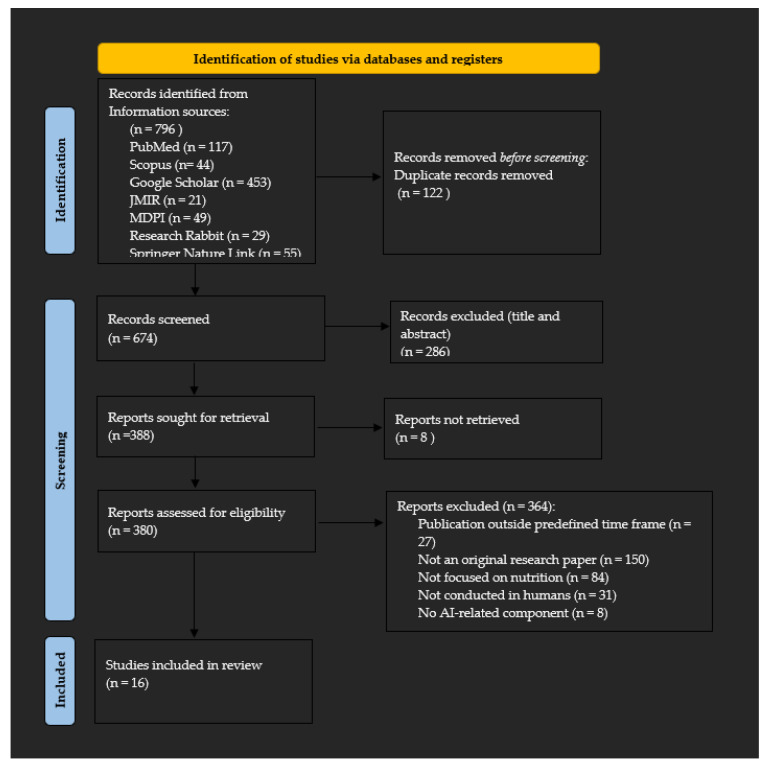
The PRISMA 2020 flow diagram illustrates the study selection process.

Of these, 364 reports were excluded for the following reasons: publication outside predefined time frame (n = 27), not original research paper (n = 150), not focused on nutrition (n = 84), not conducted in humans (n = 31), no AI-related component (n = 8), no evaluative study design (n = 60), or incomplete intervention (n = 4). Sixteen studies were included in the qualitative synthesis. The study selection process is illustrated in the PRISMA 2020 flow diagram (Figure 1). Disagreements between reviewers were resolved through discussion and consensus.

### 2.6. Data Extraction Process

Data extraction was performed independently by two reviewers using a standardized data extraction form developed a priori. Extracted information included study design, population characteristics, type of AI-based intervention, comparator (if applicable), health outcomes assessed, reported clinical impact, and level of algorithmic implementation. Discrepancies were resolved through discussion and consensus.

The selection of data items was guided by the predefined research question and PICOS framework. No assumptions were made regarding missing outcome data, and only explicitly reported study findings were extracted and synthesized. As no quantitative synthesis was conducted, summary effect measures such as risk ratios or mean differences were not calculated.

### 2.7. Post-Selection Classification of AI-Based Interventions

After completion of study selection and data extraction, an additional analytical classification process was conducted to characterize the type of artificial intelligence implemented in each included intervention. This framework was developed based on established conceptual literature on artificial intelligence and clinical decision-support systems [15,16,17]. The classification was applied retrospectively to the extracted data to enable structured and consistent differentiation of algorithmic approaches across studies.

To facilitate structured differentiation of algorithmic approaches, they were applied independently by two reviewers during data extraction, and disagreements were resolved through consensus. This analytical step did not influence study inclusion or exclusion decisions but was intended to facilitate a more nuanced interpretation of the heterogeneity in AI reporting across the selected studies (Table 2).

This classification framework was used to standardize categorization and to support subsequent analysis of algorithmic implementation across studies.

### 2.8. Risk-of-Bias Assessment

Methodological quality was assessed according to study design. Randomized controlled trials were evaluated using the Cochrane Risk of Bias 2 (RoB 2) tool [23], whereas non-randomized studies were assessed using the ROBINS-I tool [24]. Assessments were performed independently by two reviewers, and discrepancies were resolved through consensus.

This review was not prospectively registered, and no review protocol was publicly available prior to study initiation.

### 2.9. Certainty-of-Evidence Assessment

The overall certainty of evidence was not formally graded using frameworks such as GRADE. Due to heterogeneity in study design, AI implementation level, outcome definitions, and risk-of-bias assessments, a unified certainty rating across studies was not considered methodologically appropriate. Consequently, conclusions are based on narrative synthesis and qualitative interpretation of methodological rigor.

## 3. Results

Study selection

A total of 796 records were identified. After screening and eligibility assessment, 16 studies were included in the qualitative synthesis (Figure 1).

### 3.1. Description of the Selected Studies

**Figure 2:**
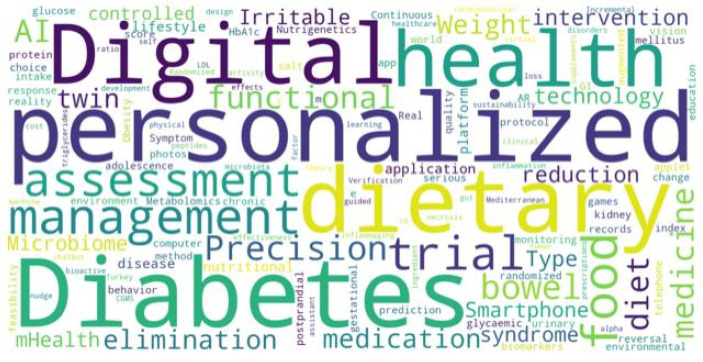
Keywords for the studies are included in this review.

The keywords that were most prevalent in the 796 studies included in this review (excluding nutrition and artificial intelligence) were personalized, diabetes, digital, and dietary. This pattern indicates that current research is focusing on personalizing nutrition through digital health technologies such as mobile health (mHealth) applications, wearable devices, telehealth platforms, and AI-driven decision-support systems, with a particular emphasis on improving diabetes care. These keywords were processed using Python 3.13, with the findings illustrated in Figure 2.

**Figure 3:**
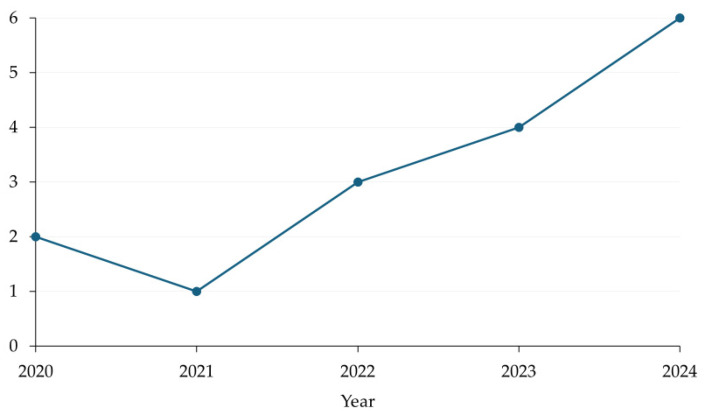
Number of selected studies per year (N = 16).

Considering that artificial intelligence remains an emerging area within mainstream nutritional applications, the temporal distribution of publications (Figure 3) demonstrates a clear upward trajectory over the study period. After a modest output in the early years, research activity increased steadily from 2022 onward, culminating in the highest concentration of publications in 2024. This pattern reflects the recent consolidation and growing research interest in AI-driven approaches within nutrition science.

**Figure 4:**
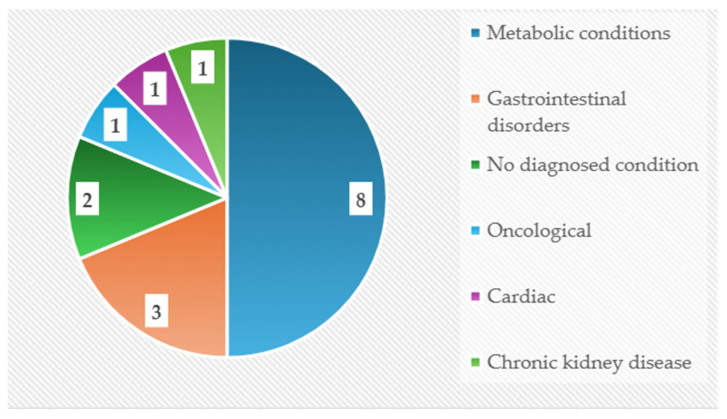
Participants’ diseases.

Of the 16 studies included, nine were randomized controlled trials [3,25,26,27,28,29,30,31] and one was a multicenter RCT [32]. One additional study was described as a clinical trial [33]. The remaining five comprised one retrospective study [34], one observational retrospective pre–post comparison [35], one cohort study [36], one retrospective cohort [37], and one single-arm intervention [38] (Figure 4).

Three studies implemented AI-assisted personalized diets based on microbiome composition to improve gastrointestinal or metabolic outcomes [27,28,33]. Two studies focused on preventing deterioration of nutritional status in at-risk populations, including pediatric post-surgical patients and hospitalized adults [25,32].

Artificial intelligence was integrated into interventions targeting a range of clinical conditions, predominantly metabolic disorders, including obesity, diabetes, dyslipidemia, and hospital-related nutritional risk [3,26,29,30,31,32,35]. Additional applications addressed gastrointestinal diseases such as irritable bowel syndrome and functional constipation [27,28,33], as well as oncology care [36], chronic kidney disease [37], and pediatric post-cardiac surgery populations [25]. In two studies, AI-driven systems were applied in individuals without diagnosed disease to support dietary optimization and lifestyle improvement [38,39,40].

**Figure 5:**
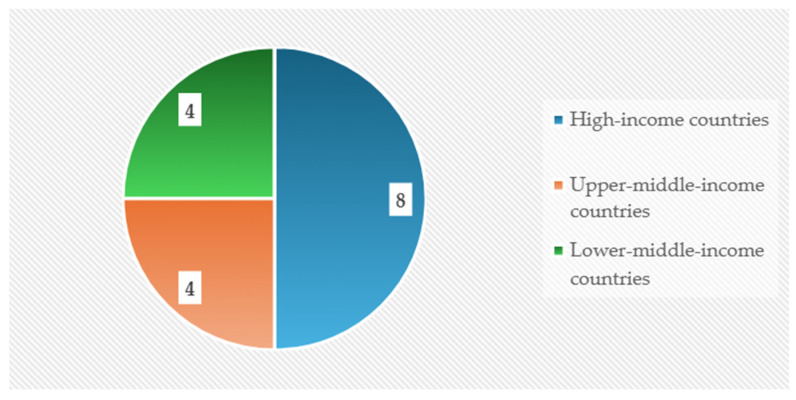
Countries’ income.

Geographically, the included studies were predominantly conducted in Asian settings, with additional contributions from North America and Oceania, while representation from Europe was limited and no eligible studies originated from Africa or Latin America. When contextualized according to the World Bank income classification [41] (Figure 5), the evidence base reflects a concentration of research in economically developed settings, with comparatively fewer investigations conducted in middle-income countries.

**Figure 6:**
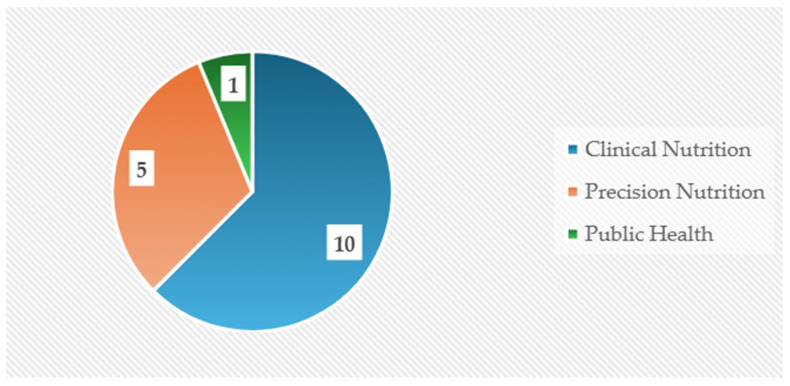
Number of studies per domain.

Although five thematic categories were predefined in the analytical framework, only three were represented among the included studies: Clinical Nutrition, Precision Nutrition, and Public Health. Notably, Clinical Nutrition concentrated on the majority of studies, followed by Precision Nutrition, while Public Health appeared marginally. The absence of the remaining two categories suggests that current implementations of AI in nutrition are still concentrated in applied clinical and individualized contexts, as illustrated in Figure 6.

Taken together, these descriptive analyses provide a contextual overview of the populations, geographic distribution, and thematic domains represented in the included studies. To further examine the characteristics and outcomes of each intervention in greater detail, a structured synthesis of the selected studies is presented in Table 3 and Table 4, summarizing study design, population, AI-based intervention, evaluated outcomes, observed clinical impact, and level of algorithmic implementation. Detailed domain-level evaluations are provided on the Supplementary Figure S1.

### 3.2. Technical Characterization of Artificial Intelligence Implementation

All 16 included studies reported the use of artificial intelligence (AI) or machine learning, with 13 explicitly referencing AI in the title and the remaining three describing it within the methods or system description. Based on the predefined operational framework, three studies were classified as Level 1 (data-driven systems), explicitly detailing adaptive machine learning or neural network models trained on longitudinal or patient-generated data. Three studies were categorized as Level 2 (knowledge-based systems), relying on expert-curated or rule-based logic without evidence of adaptive learning. Five studies were classified as Level 3 (automated digital platforms), incorporating algorithmic automation without describing adaptive model updating. The remaining five studies were assigned to Level 0 (AI declared without technical description), as insufficient methodological detail was provided to determine the nature of the AI implementation; in one case, algorithmic details were withheld due to intellectual property restrictions.

Overall, although AI was nominally declared across all studies, only a minority of studies provided sufficient methodological detail to confidently confirm adaptive machine learning implementation. For more details on this analysis, please refer to Supplementary Table S5.

Importantly, the variability in algorithmic transparency and technical reporting indicates that the term “artificial intelligence” was applied inconsistently across studies. A considerable proportion of interventions were classified as Level 0 or Level 3, reflecting either insufficient methodological detail or absence of clearly described adaptive learning mechanisms. Only a minority of studies provided explicit evidence of data-driven model training and updating. This technological heterogeneity further limits the ability to correlate AI sophistication with clinical effectiveness.

### 3.3. Risk of Bias

**RoB 2 (randomized trials):** Among the studies assessed using RoB 2, the category of **“Some concerns”** predominated across several domains, particularly those related to deviations from intended interventions and missing outcome data. Although the randomization process and outcome measurement more frequently received **low-risk** judgments, the recurrent presence of methodological concerns suggests limitations in implementation, protocol adherence, or transparency in reporting. Overall, the global risk of bias was mainly classified as “Some concerns,” with a small number of studies rated as **high risk**, indicating that effect estimates should be interpreted with caution.

**ROBINS-I (non-randomized studies):** In the studies evaluated using ROBINS-I, a greater severity of bias judgments was observed, with a substantial proportion classified as **“Serious”** and several as **“Critical,”** particularly in the domains of confounding and participant selection. Although certain domains—such as classification of interventions or measurement of outcomes—were occasionally rated as low risk, the observational nature of these designs inherently increased susceptibility to structural biases. Consequently, the overall risk of bias tended to fall within the serious or critical categories, implying that the reported effect estimates may be substantially influenced by uncontrolled factors and should be interpreted within a cautious analytical framework.

Overall, the distribution of bias judgments across study designs suggests that the current evidence base remains methodologically fragile, reinforcing the need for cautious interpretation of reported benefits.

## 4. Discussion

Our systematic review identifies emerging applications but also highlights the limited robustness of the current evidence base. Our systematic review confirms this potential but also reveals a critical gap between conceptual promises and validated, real-world applications. Of the 796 studies initially identified, only 16 met the inclusion criteria, highlighting the scarcity of empirically tested AI interventions in human populations. This mirrors the broader observation that much AI-related nutrition research remains in proof-of-concept or pilot study stages with limited translation into practice. In addition, most of the studies were conducted in small samples and showed a significant risk of bias.

Despite the growing enthusiasm surrounding artificial intelligence in nutrition, a closer examination of the included studies reveals substantial heterogeneity in methodological rigor, study design, and the depth of AI integration. Although more than half of the selected studies employed randomized controlled trial designs, many were limited by small sample sizes, short intervention periods, or narrow outcome measures, which constrain the robustness and generalizability of their findings. Retrospective and observational designs, while valuable for early-stage exploration, further limit causal inference regarding the specific contribution of AI to observed outcomes. In addition, the variability in algorithmic transparency and implementation level further complicates causal interpretation, as several interventions classified under the AI label did not provide sufficient technical detail to confirm adaptive learning mechanisms.

A critical challenge identified across studies is the difficulty in disentangling the independent effect of AI from other components of the intervention. In several cases, AI-based systems were embedded within broader behavioral or clinical support frameworks that included nutritional counseling, educational content, or clinician feedback. While these multimodal interventions frequently reported improvements in clinical or behavioral outcomes—such as weight loss, glycemic control, or dietary adherence—it remains unclear to what extent these benefits can be directly attributed to the AI component rather than to increased engagement, accountability, or intensified human support. This distinction is particularly important given that most studies did not include dismantling designs capable of isolating the independent contribution of the algorithmic component.

Furthermore, the depth and sophistication of AI implementation varied considerably. Some studies employed adaptive, data-driven models capable of learning from user inputs and dynamically updating recommendations, which aligns with contemporary definitions of artificial intelligence. In contrast, other interventions relied on algorithmic personalization based on predefined rules or limited parameter adjustments, raising questions about whether these systems truly leveraged AI or functioned primarily as advanced digital tools. This variability complicates cross-study comparison and underscores the need for clearer reporting standards regarding model architecture, learning mechanisms, and validation processes.

From a clinical perspective, the evidence for clear and sustained patient benefit remains mixed. While several studies reported statistically significant differences in intermediate outcomes favoring the use of AI, such as short-term weight loss or biomarker changes, few evaluated long-term sustainability, real-world adherence beyond study conditions, or downstream health outcomes. Additionally, user retention and engagement—critical determinants of effectiveness in digital health interventions—were inconsistently measured and rarely linked to specific AI-driven functionalities. Studies testing the efficacy of AI-based tools, as well as cost–benefit, remain to be conducted. Furthermore, the moderate-to-high risk-of-bias judgments observed across several studies reinforce the need for cautious interpretation of reported improvements.

Taken together, these findings suggest that, although AI-enabled nutrition interventions show promising potential, the current evidence base remains preliminary. Advancing the field will require more rigorous study designs capable of isolating the causal contribution of AI, standardized outcome measures that extend beyond short-term efficacy, and transparent reporting of the technological components underpinning these systems. Without such methodological advances, it will remain difficult to move from descriptive demonstrations of feasibility toward a robust, evidence-based understanding of when and how artificial intelligence meaningfully enhances nutrition care. Interventions that combine AI with behavioral strategies—such as nutritional coaching, goal-setting frameworks, or interactive education—consistently reported superior outcomes in adherence, weight reduction, and improvements in metabolic markers. This aligns with established behavior change models, which emphasize the synergy between technological tools and motivational, social, and environmental factors in driving sustainable health behaviors. Evidence from related fields shows that technology-assisted interventions without behavioral scaffolding often underperform in long-term adherence metrics.

A significant gap in the existing evidence is the absence of studies conducted in low- and middle-income countries. This geographical imbalance limits the generalizability of findings and overlooks a substantial opportunity for AI to act as an equalizer in nutrition care. AI-enabled platforms, particularly mobile-based solutions, could expand access to nutritional education, dietary monitoring, and clinical support in resource-constrained settings at a fraction of traditional intervention costs. Moreover, AI can alleviate healthcare workforce shortages by automating routine assessments, streamlining triage processes, and augmenting diagnostic decision-making through large-scale integration of lifestyle, clinical, and sociodemographic data. For this purpose, AI-based tools should be developed according to the culture, resources, and requirements of different countries and health systems.

### 4.1. Theoretical Implications

AI has been widely proposed as a transformative tool across sectors; however, its penetration into end-user care and its measurable impact remain unclear. This review provides a clearer picture: among the 796 studies identified, only 16 involved direct patient interventions, and most targeted metabolic disorders—primarily diabetes. By systematically mapping AI applications across five key domains of nutrition—Clinical nutrition, Precision nutrition, Biomarker discovery, Public health, and Food science—this review offers researchers, healthcare professionals, and industry leaders a detailed understanding of the degree of implementation, the technological approaches employed, and the populations targeted. Importantly, our findings demonstrate that, while nutrition science and AI have often evolved as separate disciplines, there is a growing body of interdisciplinary work leveraging tools from the technology sector—such as deep learning-based image recognition, wearable-integrated monitoring, and forecasting AI.

### 4.2. Practical Implications

The findings carry direct implications for technology developers, healthcare providers, policymakers, and health-sector companies. First, user-centered design emerges as a critical determinant of effectiveness: successful interventions maximized the value of patient-generated data—such as anthropometrics, dietary patterns, physical activity, and sleep—while ensuring that systems were intuitive, accessible, and motivating. Interfaces that provided clear, frequent, and personalized feedback yielded higher adherence rates. Second, explainability in AI models is essential to foster trust among both patients and healthcare providers, accuracy without interpretability risks of rejection in clinical settings. Third, the review supports the integration of AI-assisted nutrition tools into chronic disease management programs, particularly for obesity, type 2 diabetes, and metabolic syndrome, where their potential to enhance adherence and outcomes is most evident.

### 4.3. Future Directions

Ethical and regulatory considerations remain pivotal. The limited number of fully implemented clinically validated tools reflects both the nascency of the regulatory framework and ongoing concerns about data privacy, algorithmic bias, and accountability. Premature deployment without rigorous validation risks exacerbating inequities, particularly if models are trained on non-representative datasets. Therefore, future research should prioritize:

- Multi-center randomized controlled trials across diverse demographic and socioeconomic contexts.
- Integration of underutilized data streams such as metabolomics, exposomics, and geospatial food-environment mapping.
- Comprehensive cost-effectiveness analysis to inform reimbursement and adoption.
- Development of regulatory policies capable of evolving alongside technological innovation.

In summary, while the narrative surrounding AI in nutrition is one of optimism, this review demonstrates that its true potential will only be realized through deliberate, evidence-based integration into holistic nutrition care models. By combining algorithmic precision with human **expertise** and embedding AI within supportive behavioral and policy frameworks, the field can move toward equitable, scalable, and impactful nutrition interventions globally.

## 5. Conclusions

Based on this review, it appears that the application of AI-based technologies to patients is still scarce in the nutritional field. Considering the interventions included in this review, it appears that it is focused on preventing and managing disease, mainly metabolic, by either providing the patient with a dietary recommendation or prescription based on their individual needs and characteristics or by allowing patients to more readily access nutritional support, which allows them to adjust their habits and dynamically adapt their lifestyle. Not all study designs allow for a clear conclusion as to whether AI is improving care.

Nevertheless, although several studies report promising trends, the current evidence does not allow definitive conclusions regarding the independent effectiveness of AI-based systems. With rigorous methodological development, transparent reporting of algorithmic architecture, and adequately powered trials, future research may clarify whether AI integration delivers benefits beyond conventional digital or behavioral interventions.

## 6. Limitations

This systematic review presents some important limitations that should be acknowledged. The eligibility criteria reduced the number of publications to only 16 studies with technology-assisted nutritional interventions. This finding highlights a clear gap in the literature; many publications focus on theoretical proposals, technological development, or isolated validations, but few evaluate the real impact of these tools under control or semi-experimental conditions. Additionally, the heterogeneity in technological implementation and the limited reporting of algorithmic details restricted the ability to systematically compare levels of AI sophistication across interventions. The absence of meta-analysis and formal certainty grading further limits quantitative synthesis of effect magnitude.

Also, this review only focuses on human studies with specific characteristics, leaving room for further exploration of AI applications within other domains such as dietary assessment, physical activity analysis, and medical tests and procedures. This scarcity of robust evidence opens a clear avenue for future research: more empirical literature is needed with well-designed, replicable interventions evaluated across diverse population contexts.

At present, AI in nutrition should be regarded as an evolving adjunctive tool rather than a definitively validated therapeutic driver.

## Figures and Tables

**Table 1:** Eligibility Criteria According to PICOS Framework.

Element	Inclusion Criteria	Exclusion Criteria
P	Human participants (any age or clinical condition).	Animals, in vitro, simulation, or non-human studies.
I	Nutritional interventions incorporating AI components (data-driven learning, predictive modeling, adaptive decision-support).	Digital tools without algorithmic modeling; static rule-based or purely educational systems.
C	Standard care, non-AI support, or baseline values (when applicable).	Not excluded from the absence of the comparator.
O	Health-related outcomes (clinical biomarkers, nutritional parameters, disease indicators, and patient-reported outcomes).	Technical or computational metrics without health-related outcomes.
S	Original studies: randomized controlled trials or prospective/retrospective cohorts.	Reviews, meta-analysis, abstracts, editorials, protocols, or technical validations without human outcomes.

**Table 2:** Operational framework for AI implementation levels.

Level	Category	Operational Definition
Level 0	AI Declared Without Technical Description	Systems that declare the use of artificial intelligence do not provide sufficient technical details to determine whether the system is data-driven, rule-based, or automated digital.
Level 1	Data-driven systems (ML/DL)	Models trained on datasets that learn patterns through statistical optimization and adaptive parameter updating.
Level 2	Knowledge-based or rule-driven systems	Systems based on predefined expert-encoded rules or deterministic logic without adaptive learning mechanisms.
Level 3	Digital platforms with automated decision-support	Digital health tools incorporating algorithmic or automated components that do not implement adaptive machine learning models.

**Table 3:** Summary of Clinical Nutrition Studies Systematic Literature Review.

Study	Category	Design/n	Population	AI-Based Intervention	Health Outcome Evaluated	Clinical Impact Observed	AI Level
Buchan, 2024 [37]	Clinical Nutrition	Cohort; n = 3310	Cancer patients	AI-enabled nutrition support platform (INA)	QoL; engagement	Improved patient-reported QoL	Level 2
Zahid, 2023 [26]	Clinical Nutrition	RCT; n = 80	Pediatric post-cardiac surgery	AI-labelled dietary planning app	Weight; caloric; protein intake	Modest improvements vs. control	Level 3
Yanai, 2023 [40]	Clinical Nutrition	Retrospective; n = 35	Adults with CKD	ML-based salt intake estimation app	Salt intake; BMI	Short-term salt reduction	Level 3
Mosquera-Lopez, 2023 [27]	Clinical Nutrition	RCT; n = 13	Adults with T1D	Neural network-driven insulin automation	Glycemic control	Improved detection and postprandial control	Level 1
Tunali, 2024 [28]	Clinical Nutrition	RCT; n = 121	Adults with IBS	AI-assisted microbiome-based personalized diet	IBS-SSS	Greater symptom reduction vs. FODMAP	Level 0
Braga, 2024 [25]	Clinical Nutrition	RCT; n = 36	Female adolescents	AI-referenced dietary logging app	Feasibility; diet diversity	High adherence; limited dietary change	Level 3
Maher, 2020 [38]	Clinical Nutrition	Single arm; n = 31	Inactive adults	AI-enabled virtual coach	MedDiet score; weight	Modest adherence improvement	Level 2
Arslan, 2022 [29]	Clinical Nutrition	RCT; n = 45	Functional constipation	AI-assisted microbiome diet	CBMpW; QoL	Greater bowel improvement vs. control	Level 0
Pokushalov, 2024 [32]	Clinical Nutrition	RCT; n = 67	Elevated LDL-C adults	AI-guided supplement prescription	LDL-C	Greater LDL-C reduction vs. physician-guided	Level 0
Lee, 2023 [31]	Clinical Nutrition	RCT; n = 294	Adults with T2D	Deep learning-based digital health platform	HbA1c; weight	Greater reductions vs. standard care	Level 3

**Abbreviations:** RCT = Randomized Controlled Trial; QoL = Quality of Life; CKD = Chronic Kidney Disease; T1D = Type 1 Diabetes; IBS = Irritable Bowel Syndrome; IBS-SSS = Irritable Bowel Syndrome Severity Scoring System; FODMAP = Fermentable Oligosaccharides, Disaccharides, Monosaccharides and Polyols; MedDiet = Mediterranean Diet; CBMpW = Complete Bowel Movements per Week; LDL-C = Low-Density Lipoprotein Cholesterol; ML = Machine Learning.

**Table 4:** Summary of Precision Nutrition and Public Health Studies Systematic Literature Review.

Study	Category	Design/n	Population	AI-Based Intervention	Health Outcome Evaluated	Clinical Impact Observed	AI Level
Shamanna, 2021 [36]	Precision Nutrition	Retrospective; n = 463	Adults with T2D	Digital twin-guided personalized diet + monitoring	Reversal stage; HbA1c	Redistribution toward earlier reversal stages	Level 1
Aldubayan, 2022 [3]	Precision Nutrition	RCT; n = 100	Overweight/obese adults	ML-stratified personalized diet	Fat mass	No significant difference	Level 0
Karakan, 2022 [34]	Precision Nutrition	Clinical trial; n = 50	Adults with IBS-M	XGBoost microbiome-based diet	IBS-SSS	Significant symptom improvement	Level 0
Shamanna, 2020 [35]	Precision Nutrition	Retrospective; n = 64	Adults with T2D	Digital twin precision nutrition	HbA1c; HOMA-IR	Significant metabolic improvement	Level 1
Khokhar, 2024 [30]	Precision Nutrition	Multinational RCT; n = 391	Adults with obesity	AI-powered weight platform	% weight loss	Mean 14% weight reduction	Level 2
Sun, 2024 [33]	Public Health	Multicenter RCT; n = 5763	Hospitalized adults	ML-based rapid nutritional diagnostic system	Cure rate; ICER	Improved cure rate and cost-effectiveness	Level 3

**Abbreviations:** RCT = Randomized Controlled Trial; T2D = Type 2 Diabetes; HOMA-IR = Homeostatic Model Assessment of Insulin Resistance; IBS-M = Irritable Bowel Syndrome–Mixed subtype; IBS-SSS = Irritable Bowel Syndrome Severity Scoring System; ML = Machine Learning; ICER = Incremental Cost-Effectiveness Ratio.

## Data Availability

No new data were created or analyzed in this study.

## Supplementary Materials

The following supporting information can be downloaded at: https://www.mdpi.com/article/10.3390/nu18091340/s1; Table S1. Structured Boolean Search Strategy in Electronic Bibliographic Databases; Table S2. Keyword-Based Search Queries in Academic Search Engine; Table S3. Keyword-Based Search Queries in Publisher; Table S4. Citation-Network Exploration Using Seed Articles and Keyword-Initiated Mapping; Table S5. AI implementation characterization; Figure S1. Risk of Bias.

## Abbreviations

Abbreviation	Full Term
AI	Artificial Intelligence
ML	Machine Learning
DL	Deep Learning
RCT	Randomized Controlled Trial
RoB 2	Risk of Bias 2 Tool
ROBINS-I	Risk Of Bias In Non-randomized Studies—of Interventions
PICOS	Population, Intervention, Comparison, Outcomes, Study Design
HbA1c	Glycated Hemoglobin
HOMA-IR	Homeostatic Model Assessment of Insulin Resistance
QoL	Quality of Life
CKD	Chronic Kidney Disease
T1D	Type 1 Diabetes
T2D	Type 2 Diabetes
IBS	Irritable Bowel Syndrome
IBS-M	Irritable Bowel Syndrome—Mixed subtype
IBS-SSS	Irritable Bowel Syndrome Severity Scoring System
FODMAP	Fermentable Oligosaccharides, Disaccharides, Monosaccharides and Polyols
MedDiet	Mediterranean Diet
CBMpW	Complete Bowel Movements per Week
LDL-C	Low-Density Lipoprotein Cholesterol
ICER	Incremental Cost-Effectiveness Ratio
mHealth	Mobile Health
PRISMA	Preferred Reporting Items for Systematic Reviews and Meta-Analyses
GRADE	Grading of Recommendations Assessment, Development and Evaluation
