# Supplementary material for: Implementation and Applications of Artificial Intelligence in Nutrition: A Systematic Review of Use in Practice and Research
Source: Nutrients. 2026 Apr 24;18(9):1340. doi: 10.3390/nu18091340 (PMC13165132; doi:10.3390/nu18091340)
Supplement: Supplementary file 1 [file nutrients-18-01340-s001.zip › nutrients-4088547-supplementary.pdf]

Supplementary Table S1. Structured Boolean Search Strategy in Electronic Bibliographic Databases.

Search was executed between January and March 2025. No automated publication-type filters were applied at database level unless specified. Year and language restrictions were applied during screening according to PICOS criteria.

Database	Boolean Search String (verbatim)	Records Retrieved (n)
PubMed	"Clinical Nutrition" AND "artificial intelligence"	15
PubMed	"Precision Nutrition" AND "artificial intelligence"	13
PubMed	"artificial intelligence" AND "bariatric"	13
PubMed	"artificial intelligence" AND "food composition"	1
PubMed	"artificial intelligence" AND "glucose"	46
PubMed	"artificial intelligence" AND "proteins" AND "nutrition"	4
PubMed	"Artificial intelligence" AND "Nutrition" AND "Public Health"	18
PubMed	"Artificial intelligence" AND "Personalized nutrition"	2
PubMed	"Artificial Intelligence" AND "Personalized nutrition"	5
Scopus	"Artificial Intelligence" AND "Food Science"	1
Scopus	"Artificial Intelligence" AND "Nutrition"	4
Scopus	"Clinical Nutrition" AND "artificial intelligence"	2
Scopus	"Precision Nutrition" AND "artificial intelligence"	6
Scopus	"Public Health" AND "artificial intelligence"	2
Scopus	"biomarkers discovery" AND "artificial intelligence"	1

Database	Boolean Search String (verbatim)	Records Retrieved (n)
Scopus	"artificial intelligence" AND "cholesterol"	3
Scopus	"artificial intelligence" AND "dietary intervention"	7
Scopus	"artificial intelligence" AND "dietetics"	3
Scopus	"artificial intelligence" AND "glycemic"	1
Scopus	"artificial intelligence" AND "nutrigenomics"	1
Scopus	"artificial intelligence" AND "personalized nutrition"	13

Supplementary Table S2. Keyword-Based Search Queries in Academic Search Engine.

Search was executed between January and March 2025 using the platform's default relevance sorting. Results were screened sequentially in descending relevance order. The screened range per query is reported as the number of results retrieved (n). No advanced publication-type filters were applied at platform level.

Platform	Keyword-Based Query (verbatim)	Records Retrieved (n)
Google Scholar	"AI" AND "nutrition therapy" AND "oncology"	20
Google Scholar	"Artificial Intelligence" AND "Biomarkers"	4
Google Scholar	"Artificial Intelligence" AND "Eating Disorders"	1
Google Scholar	"Artificial Intelligence" AND "Nutrition"	12

Platform	Keyword-Based Query (verbatim)	Records Retrieved (n)
Google Scholar	"Artificial intelligence" AND "Biomarkers" AND "Nutrition"	28
Google Scholar	"Artificial intelligence" AND "Eating disorders"	3
Google Scholar	"Artificial intelligence" AND "Food Science" AND "Nutrition"	4
Google Scholar	"Artificial intelligence" AND "Nutrition"	33
Google Scholar	"Artificial intelligence" AND "Personalized nutrition"	6
Google Scholar	"Artificial intelligence" AND "Precision Nutrition"	8
Google Scholar	Clinical Nutrition + Artificial Intelligence	76
Google Scholar	Precision Nutrition + Artificial Intelligence	91
Google Scholar	Public Health + Nutrition + Artificial Intelligence	3
Google Scholar	Food Science + Nutrition + Artificial Intelligence	44
Google Scholar	“Inteligencia artificial” AND “Nutricion”	3
Google Scholar	“Inteligencia artificial” AND “Ciencia de los alimentos”	1

Supplementary Table S3. Keyword-Based Search Queries in Publisher.

Search was conducted between January and March 2025 using platform-native search interfaces. Results were screened in relevance order. Filters, when available, were limited to publication year; study design restrictions were applied during eligibility screening.

Publisher Platform	Search Query (verbatim)	Records Retrieved (n)
SpringerLink	"Artificial intelligence" AND "Blood glucose"	19
SpringerLink	"artificial intelligence" AND "dietary intervention"	17
SpringerLink	"artificial intelligence" AND "eating disorders"	9
SpringerLink	"artificial intelligence" AND "food labels"	3
SpringerLink	"artificial intelligence" AND "personalized nutrition"	7
JMIR	"Artificial intelligence" AND "Eating disorders"	4
JMIR	"Artificial intelligence" AND "Personalized Nutrition"	6
JMIR	"Artificial Intelligence" AND "Nutrition" AND "food science"	1
JMIR	"Artificial Intelligence" AND "Personalized nutrition"	7
MDPI	"Artificial Intelligence" AND "Nutrition"	20
MDPI	"artificial intelligence" AND "food"	21
MDPI	"artificial intelligence" AND "bariatric"	2
MDPI	"artificial intelligence" AND "diet"	2

Publisher Platform	Search Query (verbatim)	Records Retrieved (n)
MDPI	"artificial intelligence" AND "glucose"	2

Supplementary Table S4. Citation-Network Exploration Using Seed Articles and Keyword-Initiated Mapping.

Citation-network exploration was conducted between January and March 2025 using seed-based similarity mapping and keyword-initiated exploration. All identified records were manually screened.

Tool	Seed Article / Initial Node	Records Identified (n)
Connected Papers	Navigating next-gen nutrition care using artificial intelligence-assisted dietary assessment tools (scoping review)	6
Connected Papers	Revolutionizing the food industry: The transformative power of artificial intelligence – a review	4
Connected Papers	The Role of Artificial Intelligence in Nutrition Research: A Scoping Review	18
Research Rabbit	"Artificial intelligence" AND "Food Science" AND "Nutrition"	10
Research Rabbit	"Artificial intelligence" AND "Nutrition"	13
Research Rabbit	"Artificial intelligence" AND "Precision Nutrition"	6

Supplementary Table S5. AI implementation characterization

Paper	Specific AI Application	IA mentioned	Where Is It Mentioned?	Form of Mention	Specific AI Application	Clinical / Functional Use	Category	Level	Technical Justification According to Ope4rational Definition
**185 – PREVENTOMICS (Aldubayan 2022)**	Platform that classifies individuals into "metabolic clusters" using genetic and metabolomic data	YES	Methods section (p. 3)	AI declared algorithmic details not disclosed due to intellectual property restrictions	Platform that classifies individuals into "metabolic clusters" using genetic and metabolomic data	Generation of personalize d diet	AI declared without technical description	**Level 0**	Biomarker-based classification is mentioned, but no ML/DL architecture, training or adaptive updating mechanism is described.
**210 – Twin Precision Nutrition (Shamanna 2020)**	ML algorithm using CGM + food intake data to prevent glycemic spikes	YES	Methods and system description	"Machine learning algorithm" and digital twin technology	ML algorithm using CGM + food intake data to prevent glycemic spikes	Adaptive daily dietary recommend ation	Data-driven (ML)	**Level 1**	Use of a "machine learning algorithm" trained on continuous CGM data with dynamic updates is explicitly specified.
**223 – Diabetes Reversal (Shamanna 2021)**	AI-based digital twin for reversal stratification	YES	Title and keywords	"Artificial intelligence" and "Digital twin technology"	AI-based digital twin for reversal stratification	Dynamic adjustment of diet and medication	Data-driven (ML)	**Level 1**	"Digital twin" system based on individualized modeling; implies learning from longitudinal data.
**262 – IBS AI Diet (Karakan 2022)**	Algorithm optimizing diet based on microbiome	YES	Title and keywords	"Artificial intelligence-based personalized diet"	Algorithm optimizing diet based on microbiome	Personalize d diet design	AI declared without technical description	**Level 0**	Labeled as "AI-based," but no architecture, model type, or adaptive mechanism is described.
**331 – FRANI (Braga 2024)**	Automated food recognition via image analysis	YES	Title and abstract	"Artificial Intelligence-based telephone application"	Automated food recognition via image analysis	Automated dietary assessment	Digital platform with automated decision-support	**Level 3**	Food recognition automation; no description of clinical adaptive training.

**334 – Ina Virtual Dietitian (Buchan 2024)**	Conversational assistant based on expert-curated dataset	YES	Title and abstract	"Artificial Intelligence–Based Virtual Dietitian"	Conversational assistant based on expert-curated dataset	Automated nutritional counseling	Knowledge-based system	**Level 2**	Based on >100,000 curated expert interventions; no evidence of real-time adaptive learning.
**341 – Pediatric Post-Surgery App (Zahid 2023)**	Mobile app with dietary recommendations	YES	Title	"Artificial intelligence-based mobile application"	Mobile app with dietary recommendations	Nutritional monitoring	Digital platform	**Level 3**	Described as AI, but no ML model or explicit rule system described; primarily automated functionality.
**348 – CKD Salt App (Yanai 2023)**	AI-based meal image analysis	YES	Title and abstract	"Artificial Intelligence system for dietary assessment"	AI-based meal image analysis	Salt intake estimation	Automated digital platform	**Level 3**	Automated meal recognition; no clear evidence of clinical adaptive updating.
**388 – Robust Artificial Pancreas (Mosquera-Lopez 2023)**	Neural network for automatic meal detection	YES	Title	"using Artificial Intelligence"	Neural network for automatic meal detection	Automated insulin dosing	Data-driven (ML/DL)	**Level 1**	Explicit use of a "neural network model" with reported sensitivity and clinical validation.
**396 – Virtual Health Coach (Maher 2020)**	Chatbot with personalized sessions	YES	Title	"Artificially Intelligent Virtual Health Coach"	Chatbot with personalized sessions	Diet and physical activity coaching	Rule/knowledge-based system	**Level 2**	No adaptive ML described; functions as a programmed assistant with predefined personalization logic.
**399 – IBS Multicenter RCT (Tunali 2024)**	Microbiome-based AI-assisted system	YES	Title	"Artificial Intelligence-Assisted Personalized Diet"	Microbiome-based AI-assisted system	Personalized diet	AI declared without technical description	**Level 0**	Reported as "AI-assisted" without technical details on architecture or model training.
**400 – Functional Constipation (Arslan 2022)**	Soft computing system for personalized diet	YES	Title	"AI-Assisted Personalized Microbiome Modulation"	Soft computing system for personalized diet	Microbiome modulation	AI declared without technical description	**Level 0**	Mentions "soft computing system," but no ML training or deterministic rule structure described.

**458 – SureMediks (Khokhar 2024)**	Expert system with mobile app + connected scale	YES	Title and keywords	“AI-powered digital platform”	Expert system with mobile app + connected scale	Personalize d weight managemen t	Knowledge-based system	**Level 2**	Explicit use of an “Expert system”; corresponds to deterministic logic without adaptive learning.
**496 – Integrated T2D Platform (Lee 2023)**	AI-driven dietary management with image recognition	YES	Title and article highlights	“AI-Based Dietary Management”	AI-driven dietary management with image recognition	Glycemic control	Automated digital platform	**Level 3**	Automates dietary input via photography; no longitudinal adaptive ML model described.
**559 – GenAIS LDL Study (Pokushalov 2024)**	AI-guided personalized supplement prescription	YES	Title	“AI-Guided Dietary Supplement Prescriptions”	AI-guided personalized supplement prescription	LDL reduction	AI declared without technical description	**Level 0**	Labeled as “AI-guided,” but architecture and learning mechanism are not detailed.
**620 – AI Nutritional Diagnostic System (Sun 2024)**	AI system for hospital nutritional diagnosis	YES	Title	“Artificial Intelligence (AI)-based rapid nutritional diagnostic system”	AI system for hospital nutritional diagnosis	Automated screening	Automated digital platform	**Level 3**	Rapid evaluation system; no explicit adaptive ML model reported.

Supplementary Figure S1 — Risk of Bias

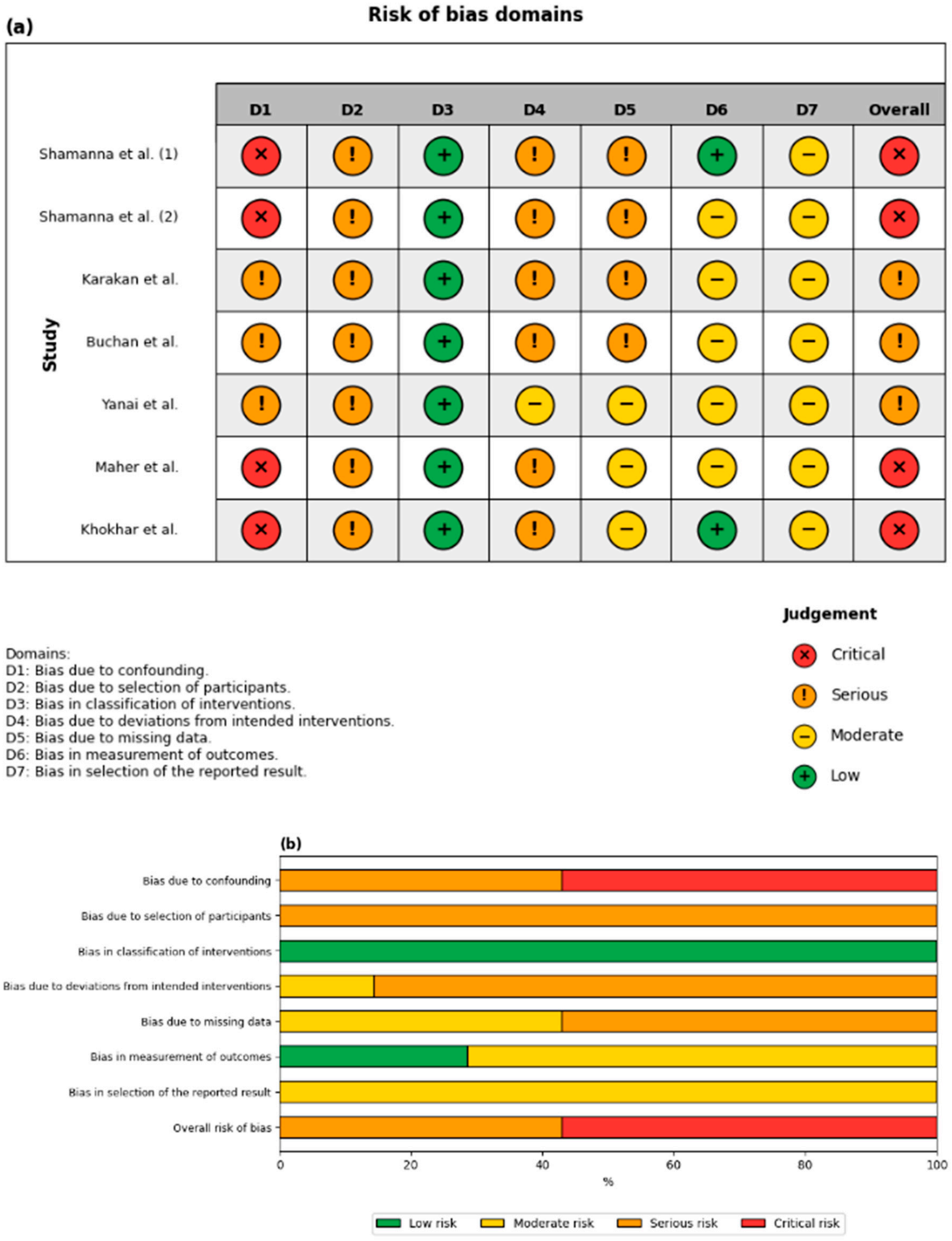

S1a. Risk-of-bias analysis performed with ROBINS-I (Risk of Bias in Non-randomized Studies-of Interventions), Cochrane.

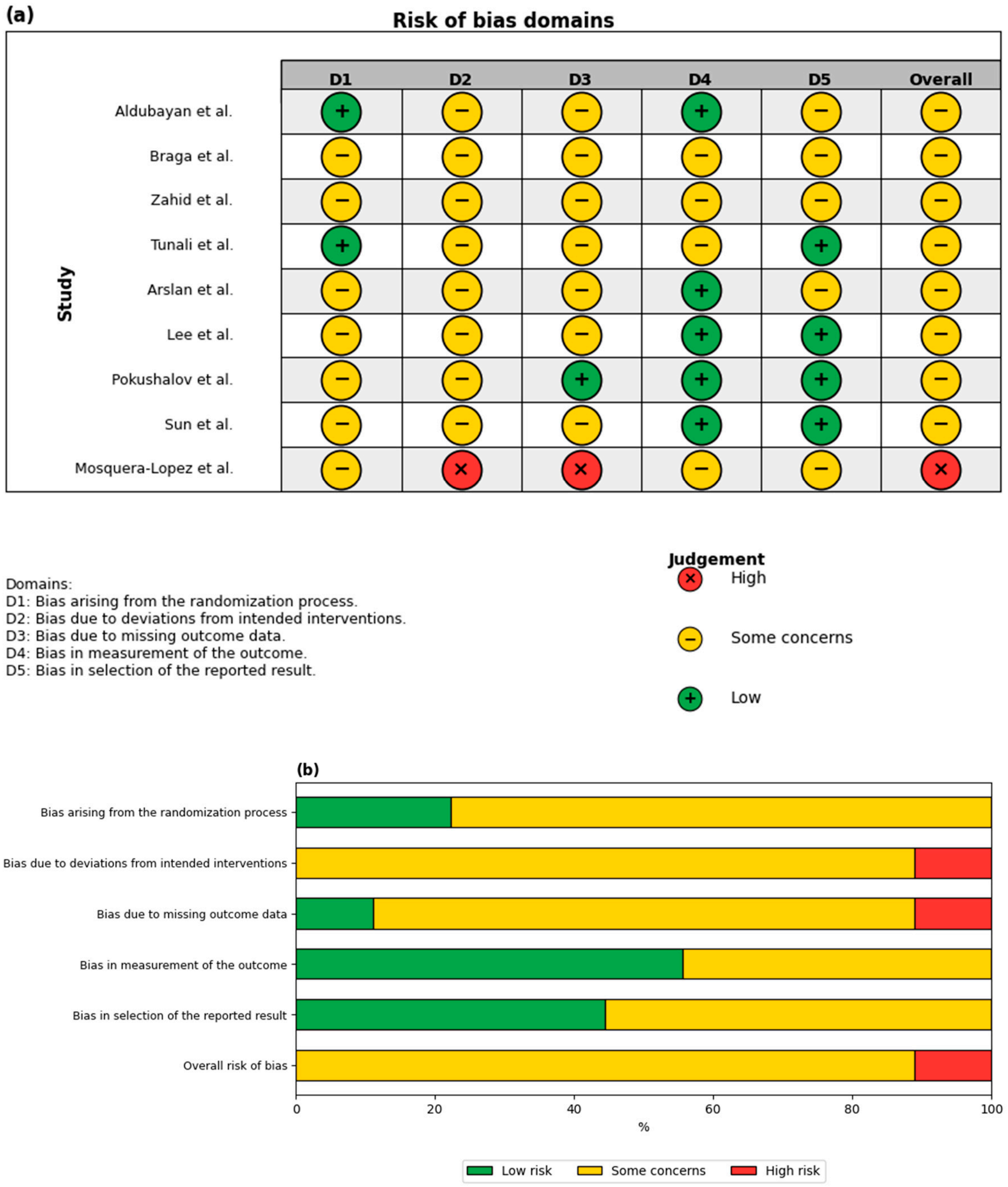

S1b. Cochrane risk-of-bias tool for randomized trials (RoB 2.0)
